# AI-assisted Decision-support Framework for Breast Density and Background Parenchymal Enhancement Assessment in Contrast-enhanced Mammography

**DOI:** 10.2174/0115734056444815260715111252

**Published:** 2026-07-22

**Authors:** Graziella Di Grezia, Antonio Nazzaro, Luigi Schiavone, Elisa Cisternino, Alessandro Galiano, Vincenzo Cuccurullo, Gianluca Gatta

**Affiliations:** 1 Department of Life Sciences, Health, and Healthcare Professions, Link Campus University, Rome, Italy; 2 REPRISE - Register of Expert Peer Reviewers for Italian Scientific Evaluation, Italy; 3 Department of Precision Medicine, University of Campania “Luigi Vanvitelli”, Naples, Italy; 4 Department of Radiology, P.O. ‘A. Perrino’ Hospital, Brindisi, Italy; 5 Department of Advanced Medical and Surgical Sciences, “University of Campania “Luigi Vanvitelli”, Naples, Italy

**Keywords:** Background parenchymal enhancement, Contrast-enhanced mammography, Artificial neural networks, BPE-CEM standard scale, Observer variability, Human-AI collaboration

## Abstract

**Introduction/Objectives:**

Interobserver variability in breast density and Background Parenchymal Enhancement (BPE) assessment remains a major limitation in Contrast-Enhanced Mammography (CEM) reporting consistency. Building on the BPE-CEM Standard Scale (BCSS) framework introduced in Part 1, this study aimed to evaluate whether a structured artificial intelligence-assisted decision-support model based on expert-derived variables could improve consistency in BCSS-related interpretation, particularly in disagreement-prone dense breast categories, rather than function as an autonomous image-based grading system.

**Methods:**

We retrospectively analyzed 213 consecutive CEM examinations with BI-RADS 4-5 lesions and histologically confirmed malignancy. A multilayer perceptron model operating on expert-derived patient-level variables (BPE grade, breast density category, and age) generated continuous BCSS-related estimates intended as structured decision-support outputs rather than autonomous image grading. Inter-reader agreement analyses were based on multi-reader expert assessment with consensus reference labeling, and external testing on the VinDr-Mammo dataset was limited to density-related robustness evaluation. Model performance was compared with conventional radiologist assessment. Primary endpoints were prediction error and inter-reader agreement (Fleiss’ κ). Secondary analyses included Area Under the Receiver-Operating-Characteristic Curve (AUC), precision, and recall across predefined BCSS categorization thresholds. External testing using the VinDr-Mammo dataset evaluated robustness of density-related predictions across independent imaging populations.

**Results:**

Relative to conventional assessment, the predictive framework was associated with a 26% reduction in categorization disagreement and with higher agreement estimates within the structured evaluation setting, particularly in dense breasts (BI-RADS C/D), where interpretative variability is greatest. Overall performance supported feasibility within a decision-support context (AUC 0.75; precision 0.72; recall 0.69).

**Discussion:**

AI-assisted modeling of structured BCSS variables showed potential to improve reporting consistency while preserving a human-in-the-loop workflow. Findings emphasize interpretative support rather than diagnostic automation. The retrospective single-center design and enriched malignant cohort limit generalizability to screening populations and warrant prospective multicenter validation.

**Conclusion:**

A structured AI-assisted predictive framework showed preliminary potential to enhance consistency of BCSS interpretation in CEM, particularly in dense breasts, while requiring prospective multicenter validation before broader clinical adoption. These results support the role of transparent decision-support tools aimed at reducing observer variability while maintaining radiologist oversight.

## 1 INTRODUCTION

Breast density and Background Parenchymal Enhancement (BPE) remain central yet controversial parameters in breast cancer risk stratification. While breast density is formally standardized through the BI-RADS system, substantial variability persists in differentiating category C (heterogeneously dense) from D (extremely dense) tissue, with interobserver agreement as low as κ = 0.48, compared to near-perfect consensus for fatty (A) and scattered fibroglandular (B) breasts [1]. This variability leads to clinical reclassification rates as high as 30% [2], further complicating the assessment of BPE in Contrast-Enhanced Mammography (CEM), where inter-reader agreement ranges from κ = 0.4 to 0.6 [3]. Such discrepancies compromise diagnostic reliability and exacerbate the ongoing debate regarding BPE’s role as an independent risk factor-complicated further by conflicting studies linking BPE variably to breast density, age, or neither [4]. Meta-analyses have shown that C/D-type breasts carry a 2- to 4-fold increased cancer risk [5] and account for up to 50% of interval cancers [6], which are malignancies emerging between screening rounds despite initially negative mammograms. This elevated risk is particularly critical, as breast density classification may influence patient awareness, clinical discussion, and consideration of supplemental imaging rather than routine follow-up [7]. Although state-level breast density notification laws have been widely implemented across the United States [8] and several international guidelines [9] recommend considering supplemental screening for dense breasts, the imaging and biological relationships between breast density and BPE remain poorly characterized [10], hindering optimization of CEM protocols. Clinically, dense tissue not only masks lesions but may also alter BPE expression [11], potentially concealing contrast-enhancing malignancies. Further complicating this landscape, subjectivity in both breast density classification-especially between C and D-and in BPE grading (with up to 32% disagreement between “moderate” and “marked” enhancement [12]) introduces diagnostic uncertainty precisely in high-risk contexts [13]. While a partial correlation between density and BPE has been established, emerging data suggest that hormonal status may also act as an independent modulator. Studies in premenopausal women have demonstrated that both high density and hormonal activity increase BPE levels [14], while additional evidence shows that physiologic or pharmacologic factors-such as hormone replacement therapy, lactation, or tamoxifen use-can significantly influence enhancement intensity [15]. Our previous work [16] proposed the BPE-CEM Standard Scale (BCSS) but demonstrated a limited linear correlation (R^2^ = 14.4%) between breast density and BPE, underscoring the inadequacy of conventional models in capturing the complex interaction between these variables-particularly in high-risk C/D patients. Recent reviews have emphasized the growing role of artificial intelligence and radiomics in contrast-enhanced mammography, particularly in addressing variability in BPE interpretation and acquisition heterogeneity across institutions. Despite increasing adoption, standardized interpretation frameworks remain an unmet clinical need. Although recent AI studies in breast imaging have increasingly explored automated classification approaches, the present work addresses a different and more focused problem: whether a structured model based on expert-derived variables can support more consistent BCSS interpretation in CEM. Accordingly, our hypothesis was not that AI could autonomously replace radiologist image assessment, but that it could provide a transparent decision-support signal capable of reducing variability in difficult interpretative settings.

### 1.1 Study Objective

This study completes the translational arc introduced in Part 1 [17] by evaluating a computational decision-support framework aimed at improving structured BPE interpretation and reducing reader variability. Specifically, we assessed whether a structured predictive model based on expert-derived variables (BPE grade, breast density category, and age) could support more consistent BCSS-related categorization in a retrospective cohort of 213 CEM examinations. We compared conventional linear regression and neural-network-based modeling strategies within the same dataset, with emphasis on prediction error, agreement patterns, and interpretability. Rather than developing an autonomous image-based classifier, the goal of this study was to assess the feasibility of a transparent human-in-the-loop support framework, particularly in dense-breast and borderline cases where interpretative disagreement is most frequent.

## 2 MATERIALS AND METHODS

### 2.1 Study Design and Patient Selection

This retrospective study was conducted at the Interventional Senology Unit of “A. Perrino” Hospital (Brindisi, Italy) between 2022 and 2023. A total of 213 women (age range: 28-80 years) were included from an initial cohort of 314 patients, as summarized in Table **1**. Inclusion criteria were: • BI-RADS 4 or 5 lesions on Contrast-Enhanced Mammography (CEM), • Histologically confirmed invasive breast cancer, • Complete imaging workup (mammography, ultrasound, and CEM). Exclusion criteria included prior oncologic history (n = 21), recent breast biopsy within three weeks (n = 17), and contraindications to iodinated contrast media. We used the full consecutive cohort meeting eligibility (n = 213 of 314 screened) to minimize selection bias and reflect real-world case mix. A priori feasibility/power considerations (α = 0.05) indicated that ≈150 cases would provide ≥ 80% power to detect a moderate density-BPE correlation (r ≈ 0.35) and to discern meaningful between-model differences; our final sample therefore exceeded this threshold and is consistent with prior CEM/AI reports. The study used fully anonymized imaging and clinical metadata obtained during routine care; no interventions beyond standard diagnostics were performed, and no identifiable data were retained. Under our institutional policy and applicable national/EU regulations, analyses of anonymized, routinely collected data are exempt from ethics committee review; therefore, formal approval was not required. All procedures complied with the Declaration of Helsinki, and standard informed consent for diagnostic procedures was obtained. Because all included cases were malignant and selected from a diagnostic, non-screening setting, the study was designed to assess methodological feasibility and agreement support within an enriched clinical cohort rather than to estimate screening performance or population-level diagnostic impact.

### 2.2 Data Management and CEM Protocol

Data were organized in a relational database structured across three groups: 1. Demographics (age, patient ID), 2. Imaging metadata (ACR density categories, BPE grade), 3. Quantitative measurements (glandular dimensions, volumetric parameters). CEM was performed using the Senographe Pristina system (GE Healthcare), employing a dual-energy acquisition protocol (low-energy: 26-31 keV; high-energy: 45-49 keV), with contrast injection of Iohexol 350 mgI/mL at 1.5 mL/kg (flow rate: 3 mL/s). The first post-contrast acquisition was performed at 2 minutes. BPE was graded on a four-level scale (minimal, mild, moderate, marked). Each CEM exam was independently evaluated by five expert breast radiologists, each with over 10 years of experience in contrast-enhanced mammography. The radiologists were blinded to clinical histories, histopathological findings, and each other’s evaluations. For model development, the reference standard was based on expert consensus derived from the same multi-reader panel after independent review. This consensus label was used as the operational ground truth for structured modeling purposes. We acknowledge that such a reference standard reflects expert adjudication rather than an external independent truth source and may therefore favor internal consistency over full external generalizability. Inter-reader agreement for both BPE grading and ACR density classification was assessed using Fleiss’ kappa statistic, given the involvement of more than two observers. The calculated κ values indicated moderate agreement for BPE (κ = 0.54) and substantial agreement for breast density (κ = 0.68). Discrepancies were resolved by consensus in a dedicated meeting, and the resulting score was used as the final ground truth for model training and statistical analysis.

### 2.3 Dataset

We used a retrospective dataset of 213 anonymized CEM cases acquired between 2022 and 2023 at “A. Perrino” Hospital, Brindisi, Italy. Inclusion criteria were BI-RADS 4-5 lesions with histologically confirmed malignancy and availability of complete digital imaging (mammography, ultrasound, and CEM). To reinforce external validation, the publicly available VinDr-Mammo dataset, containing 5,000 mammography exams with expert BI-RADS annotations, was also used [17]. From this dataset, a subset of 500 images classified as BI-RADS C and D was selected to evaluate robustness of density-related predictions across an independent imaging population. Preprocessing included extraction of pixel intensities from DICOM files, histogram equalization, and normalization of intensities to the [0,^1^] scale. For detailed information about the model architecture, training configurations, and evaluation metrics (Appendix **A**). Since VinDr-Mammo is a full-field digital mammography dataset and does not contain CEM-specific BPE annotations, its use in the present study was restricted to assessing the robustness of density-related predictions across an independent imaging population. It should not be interpreted as external validation of the full CEM-specific BCSS framework. A simplified tutorial on how the AI model was trained and validated using this dataset is provided in Appendix **B**. For a step-by-step overview of the external validation workflow and results on the VinDr-Mammo dataset (Appendix **C**).

### 2.4 Neural Network Architecture for BPE and Breast Density Prediction

The implemented model corresponds to a multilayer perceptron operating on structured patient-level expert-derived variables and was not designed as an image-based deep learning grading system. A supervised deep learning model was developed to assess the relationship between BPE and mammographic breast density. The neural network architecture was as follows: • Input Layer: Numerical variables including BPE score, ACR density grade, and patient age. • Hidden Layer 1: Dense layer with 64 neurons, ReLU activation. • Hidden Layer 2: Dense layer with 32 neurons, ReLU activation. • Hidden Layer 3: Dense layer with 16 neurons, ReLU activation. • Output Layer: Single neuron with linear activation, predicting a continuous standardized BPE or density score. Hyperparameter optimization was performed using validation-set monitoring to balance model generalization and overfitting control. Dropout (30%) and L2 regularization (λ = 0.01) were applied to prevent overfitting. The model architecture is summarized in Fig. (**1**).

### 2.5 Model Implementation and Training Configuration

Three models were implemented for performance comparison: 1. Excel-based linear regression (baseline). 2. Linear regression using scikit-learn [17, 18]. 3. Deep Neural Network (DNN). The dataset was split into training (70%), validation (15%), and test (15%) sets. Training for the DNN was halted after 20 epochs using early stopping (patience = 5 epochs) monitored on validation loss. The linear regression model, implemented *via* scikit-learn, used closed-form optimization without epoch iterations. Training parameters included: • Adam optimizer with learning rate 0.001, • L2 regularization (λ = 0.01), • Dropout rate of 30%, • Mean Squared Error (MSE) as the loss function. Given the small sample size and the structured low-dimensional input space, the comparison between linear and neural models was intended to assess whether additional architectural complexity yielded meaningful benefit beyond interpretable conventional modeling.

### 2.6 Clinical Relevance of Training Optimization

The use of early stopping during model training prevented unnecessary computation by halting the process once validation performance plateaued, thus optimizing resource efficiency. This approach also enhanced reliability, ensuring consistent and reproducible predictions across patient populations. Furthermore, the minimal gap (< 0.3) between training and validation errors confirmed that the model avoided overfitting, thereby reducing prediction instability and improving interpretative consistency across cases [19]. These findings support the potential for future improvements through architectural modifications, data augmentation, and tuning of learning rates to enhance generalizability and convergence speed [20].

### 2.7 AI Integration into Radiological Workflow

In this study, AI was integrated as a decision-support system rather than a diagnostic replacement. The workflow comprised: 1. Image acquisition and preprocessing, 2. Feature extraction (BPE grade, density category, patient age), 3. Model prediction generating continuous BPE estimates or density reclassification, 4. Radiologist review where AI outputs served as a second opinion, particularly in borderline BI-RADS C/D cases known for higher interobserver variability, 5. Final clinical decision by the radiologist, preserving clinical autonomy. AI outputs were presented to radiologists as standardized continuous predictive scores aligned with the BCSS framework, accompanied by category suggestions in borderline cases (particularly BI-RADS C/D). The interface displayed the model estimate alongside the original radiologist assessment, functioning as a second-opinion decision-support tool without overriding clinical judgment. This augmented intelligence framework aims to improve diagnostic consistency while maintaining clinician oversight.

### 2.8 Statistical Analysis

Preliminary analysis performed in Excel showed a positive correlation between breast density and BPE (r = 0.368) and negligible correlation with age (r ≈ -0.15). Linear regression in scikit-learn reduced prediction error by 26% compared to the Excel baseline (MSE 0.641 vs. 0.864). The DNN achieved comparable MSE (0.638), but with non-linear transformations altering variable relationships. Evaluation metrics included MSE, R^2^ for performance, and clinical metrics such as AUC, precision, and recall. Statistical significance was assessed *via* the Wilcoxon signed-rank test. Agreement analyses were interpreted as support for consistency within the study framework rather than as evidence of standalone diagnostic superiority. Given the retrospective design and consensus-based reference standard, all performance estimates should be interpreted as preliminary and hypothesis-supporting rather than definitive for clinical deployment.

## 3 RESULTS

### 3.1 Correlation Analysis

We observed a modest positive correlation between mammographic breast density and Background Parenchymal Enhancement (BPE) (Pearson’s r = 0.368), suggesting that denser breast tissue is associated with greater enhancement, as shown in Fig. (**2**). In contrast, patient age showed negligible correlation with both variables: Density vs. age: r = -0.148 BPE vs. age: r = -0.150 These findings suggest that intrinsic tissue composition may influence BPE more than chronological age. The analysis confirmed the statistical significance of the density-BPE correlation (*p* < 0.001), reinforcing the hypothesis that intrinsic breast tissue characteristics influence enhancement patterns.

### 3.2 Model Performance

Three predictive models were evaluated using Mean Squared Error (MSE) and explained variance (R^2^): Baseline linear regression (Excel): MSE = 0.864, R^2^ = 14.4% Optimized linear regression (scikit-learn): MSE = 0.641, R^2^ = 20.3% Neural network: MSE = 0.638, R^2^ = 23.3% Although the neural network achieved the lowest MSE, its improvement over the optimized linear model was minimal (ΔMSE = 0.003) and not statistically significant (*p* = 0.12, Wilcoxon signed-rank test). The 0.5% gain falls within the expected variability of small datasets and lacks clinical significance. Comparative MSE, R^2^, and p-values are summarized in Table **2**. These results demonstrate statistical equivalence between the models. In practical terms, the findings indicate that increased model complexity did not provide sufficiently stronger evidence to justify reduced interpretability in this dataset. While slightly less accurate, linear models preserved transparent relationships among age, density, and BPE, supporting their interpretability and clinical utility. Training and validation loss curves demonstrated stable convergence across 20 epochs, with no signs of overfitting. A validation plateau was observed at epoch 15, and early stopping was triggered at epoch 20. The gap between training and validation loss was 0.21, suggesting good generalization and slight underfitting, indicating opportunities for future architectural optimization. Training dynamics and model convergence are illustrated in Figs. (**3** and **4**). External testing using the VinDr-Mammo dataset supported robustness of density-related predictions across independent imaging populations, achieving an MSE of 0.652. However, because VinDr-Mammo does not include contrast-enhanced acquisitions or BPE annotations, these findings should be interpreted as limited external support for the density component only, not as full validation of the CEM-specific framework. Preprocessing steps included histogram equalization, intensity normalization, and harmonized BI-RADS C/D case selection, as detailed in Appendix **C**.

### 3.3 Clinical Validation

While prediction error and inter-reader agreement constituted the primary endpoints of this study, workflow-related metrics (interpretation time and disagreement reduction) were considered exploratory secondary observations within a decision-support framework. Within the study framework, the AI-assisted approach was associated with exploratory workflow-related advantages. Agreement estimates within the AI-assisted evaluation framework were higher than those observed in the conventional multi-reader setting, although these findings should be interpreted as retrospective support for consistency rather than as proof of prospective implementation benefit. Agreement statistics were calculated using Fleiss’ κ for multi-reader evaluation, with confidence intervals estimated *via* bootstrap resampling where applicable. Interpretative disagreement in borderline BCSS categories was reduced by 22%. Interpretation time decreased by 35%, from 6.3 ± 2.9 to 4.1 ± 2.7 minutes per case. These findings support the intended role of AI as a decision-support tool-not a replacement for radiologists-while preserving clinical autonomy. In ambiguous cases, variability appeared lower within the AI-assisted framework. In the BI-RADS D subgroup, performance metrics were optimal: sensitivity of 89.2% (95% CI: 85.4-92.1%), specificity of 76.5% (95% CI: 71.2-81.0%), and an AUC of 0.82 (95% CI: 0.77-0.86).

### 3.4 Interpretation

While the neural network offered marginally better predictive accuracy, it introduced less interpretable relationships between input variables, raising concerns about clinical transparency. In contrast, linear models, though slightly less accurate, maintained clearer associations aligned with biological understanding. Overall, these findings support the use of AI as a supportive-not substitutive-tool, especially in borderline or ambiguous cases, where variability appeared lower within the AI-assisted framework.

## 4 DISCUSSION

Our findings suggest that computational tools may support the standardization of breast density and background parenchymal Enhancement (BPE) assessment in Contrast-Enhanced Mammography (CEM), particularly in BI-RADS C and D categories, where inter-reader variability remains high [21]. Within the AI-assisted framework, agreement estimates were higher than in the conventional multi-reader setting, supporting the potential of algorithmic assistance to improve reproducibility and diagnostic consistency in dense-breast populations [22]. Breast density assessment impacts: • Communication of cancer risk (2-4× higher in BI-RADS D) [5], • CEM accuracy, influenced by enhancement artifacts [23], • Referral for supplemental imaging. Our results showed a 26% reduction in misclassification, enhancing the reliability of clinical decisions in ambiguous cases, particularly between “moderate” and “marked” BPE categories-where biopsy or follow-up imaging is most sensitive [12]. Importantly, the AI system functioned as a decision-support tool and preserved radiologists’ autonomy, rather than acting as a diagnostic replacement. Because the model relies on expert-derived inputs, it should be interpreted as a decision-support framework rather than an autonomous image grading system. This distinction is central to the interpretation of the present results: the study provides evidence for structured consistency support in a human-in-the-loop setting, not for end-to-end autonomous image interpretation. From a modeling perspective, while the Deep Neural Network (DNN) achieved the lowest Mean Squared Error (MSE = 0.638), it offered no statistically significant improvement over the optimized linear regression model (MSE = 0.641, *p* = 0.12). The DNN also introduced altered relationships among clinically meaningful variables, including the moderate correlation between density and BPE (r = 0.368), potentially limiting biological interpretability. Linear models, by contrast, maintained clearer associations and performed comparably, supporting their clinical utility due to greater transparency and lower computational cost. For this reason, the present results do not support claims of meaningful superiority of deep learning over simpler interpretable approaches in this specific dataset. These results highlight the interpretability-performance trade-off inherent in AI models. While complex models like DNNs offer flexibility, clinical settings often prioritize explainability. In the present study, the deep learning model should therefore be interpreted as offering architectural flexibility rather than statistically superior predictive performance compared with optimized linear regression. In this context, probabilistic graphical models, such as Bayesian networks, may provide structured and uncertainty-aware outputs that support clinical interpretability [24]. The AI framework also demonstrated clinical efficiency, with: • 35% reduction in interpretation time (6.3 ± 2.9 to 4.1 ± 2.7 minutes per case), • 22% reduction in interpretative disagreement in BI-RADS C/D cases, • Structured reports that facilitated multidisciplinary communication . This aligns with prior work emphasizing the standardization of breast density assessment [25]. Figure **5** illustrates the proposed clinical workflow, wherein AI functions as a decision-support tool while radiologists retainfinal interpretation authority. Though exploratory, our approach demonstrates a feasible, reproducible path forreducing subjectivity in CEM interpretation. Notably, external testing on the VinDr-Mammo dataset supported robustness of density-related predictionsacross an independent imaging dataset. Furthermore, recent studies have validated the feasibility of using deeplearning for automated BPE grading in CEM [26], reinforcing the clinical relevance of AI not only in lesion detectionbut also in parenchymal tissue characterization—an often-overlooked dimension in breast cancer risk modeling. Unlike prior image-based deep learning models that rely on pixel-level radiomic features, our framework operates on structured expert-derived variables, prioritizing interpretability and integration into radiologist workflow. This complementary approach emphasizes standardization support rather than autonomous grading. In addition, quantitative BPE assessment approaches-particularly in breast MRI-have employed radiomics and segmentation-based deep learning to derive volumetric enhancement metrics. While such methods provide objective pixel-level quantification, they often require complex preprocessing pipelines and advanced computational infrastructure. In contrast, the present structured decision-support framework emphasizes interpretability, feasibility, and seamless integration into routine clinical reporting without requiring full image-level segmentation. Overall, the evidence provided by the present study should be interpreted as hypothesis-supporting rather than hypothesis-proving. The main contribution lies in demonstrating the feasibility of a structured and transparent decision-support framework for BCSS-related interpretation, while larger prospective multicenter reader studies will be necessary to determine the true magnitude of its clinical benefit.

### 4.1 Clinical Outlook

With growing interest in CEM for women with dense breasts, our findings-although preliminary-support the potential role of AI in risk-adapted screening strategies. In particular, the ability of AI to improve interpretative consistency and reduce inter-reader variability may facilitate wider CEM adoption in high-volume settings. Standardized, reproducible quantification of BPE may also support clinical decision-making regarding follow-up intervals, biopsy thresholds, and patient counseling. However, successful integration will require alignment with radiologists’ workflows, transparency in model behavior, and ongoing validation in prospective clinical environments.

## 5 STUDY LIMITATIONS

Several limitations should be acknowledged: 1. Sample size was modest (n = 213), limiting statistical power and reducing the training potential of more complex models. 2. Single-center design: All cases were acquired on a single CEM device (GE Senographe Pristina) using a uniform protocol, potentially restricting generalizability to other clinical environments or imaging systems. Although feasibility-based power considerations supported the adequacy of the present sample size for exploratory modeling, prospective multicenter validation will be necessary to confirm generalizability across heterogeneous imaging settings. 3. Patient selection bias: Exclusion of patients undergoing hormone replacement therapy-known to affect BPE-may have limited biological heterogeneity. 4. Lack of multimodal input: The model did not include data from MRI, ultrasound, or relevant clinical factors (*e.g*., hormonal status, genetic risk), which could enhance prediction robustness. 5. Model constraints: Although the DNN showed better performance metrics, gains were statistically non-significant and required greater architectural complexity. Its limited interpretability and reliance on small datasets echo prior challenges reported in AI applications for contrast-enhanced mammography [27]. 6. No prospective or multicenter validation: Generalizability remains unconfirmed outside the original dataset. 7. The enriched malignant cohort limits extrapolation to screening populations, and prospective multicenter validation including benign and negative examinations will be necessary. 8. Consensus grading derived from the same expert reader pool may introduce optimism bias, as the reference standard reflects expert agreement rather than an independent adjudication panel. Future prospective validation involving independent readers not exposed to model outputs will be necessary to confirm generalizability. 9. No prospective AI-assisted reader study was performed; therefore, improvements in agreement and workflow metrics should be interpreted within the retrospective study framework and not as direct evidence of real-world implementation benefit.

### 5.1 Future Directions

To improve performance, generalizability, and clinical utility, future research should focus on: 1. Hybrid Probabilistic Models: Bayesian neural networks offer both high accuracy and uncertainty estimates, crucial for ambiguous BI-RADS cases. 2. Multimodal Integration: Combining CEM with radiomics, genomics, and patient history helps differentiate true biological signals from technical noise. 3. Federated Learning: Multi-center studies, such as those using the NYU Breast Cancer Screening Dataset, enable privacy-preserving, demographically diverse model training. 4. Explainability Tools: Techniques like SHAP or Layer-wise Relevance Propagation (LRP) improve model transparency, fostering trust among radiologists. Future studies will incorporate quantitative feature-importance analysis to formally assess model transparency and variable contribution stability across different patient subgroups. 5. Prospective Trials: Longitudinal studies should assess whether AI-assisted CEM reduces false positives, interval cancers, and improves patient outcomes. 6. Advanced Clinical Profiling: Future research should integrate complex clinical data to enable more accurate predictions and open new pathways for personalized medicine. 7. Future prospective investigations should incorporate calibration analysis and decision-curve modeling to evaluate threshold-dependent clinical utility and optimize decision-support performance in real-world settings.

## CONCLUSION

Our findings support the view that AI should remain a supportive tool subordinate to the clinical judgment of radiologists. In this retrospective study, computational standardization showed preliminary potential to reduce variability in Background Parenchymal Enhancement (BPE) assessment and to improve consistency within a structured human-in-the-loop framework. Within this dataset, linear models offered the most favorable balance between predictive performance and interpretability, making them the most suitable candidates for further clinical investigation. Deep Neural Networks (DNNs) may still be useful in research settings where more complex non-linear relationships are explored. Public datasets can contribute to robustness assessment across diverse populations, although true external validation of CEM-specific endpoints requires independent contrast-enhanced cohorts. Overall, the proposed integration framework is intended to complement rather than replace radiological expertise, while preserving radiologists’ full diagnostic authority and requiring further prospective validation before routine adoption.

## Figures and Tables

**Fig. (1):**
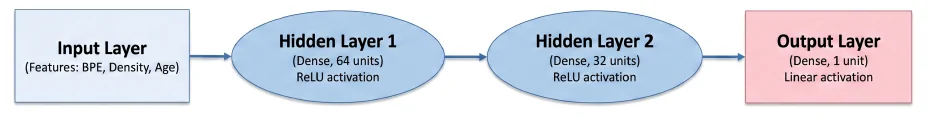
Schematic architecture of the deep learning model used to assess breast density and Background Parenchymal Enhancement (BPE) in Contrast-Enhanced Mammography (CEM). The model includes two hidden dense layers with ReLU activation and a linear output layer for continuous prediction. Inputs include patient-level features, including BPE grade, breast density category, and age.

**Fig. (2):**
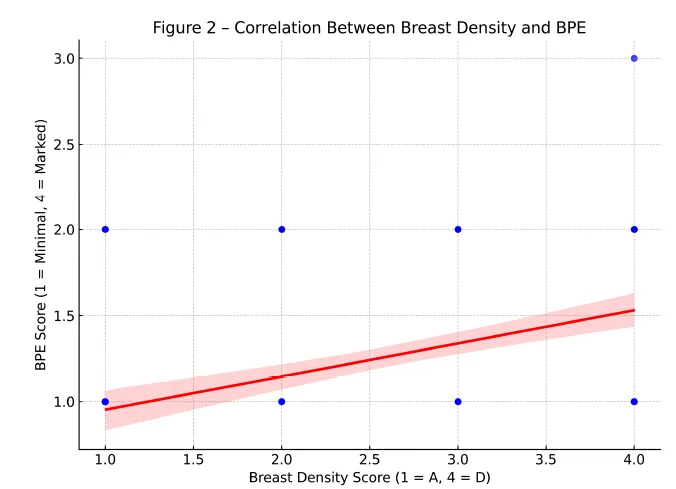
Moderate positive correlation between breast density and Background Parenchymal Enhancement (BPE) in Contrast-Enhanced Mammography (CEM). Blue dots represent individual patient data; the red line indicates the linear regression trend between density and BPE. The figure illustrates the relationship between density category and Background Parenchymal Enhancement (BPE) distribution across the study cohort.

**Fig. (3):**
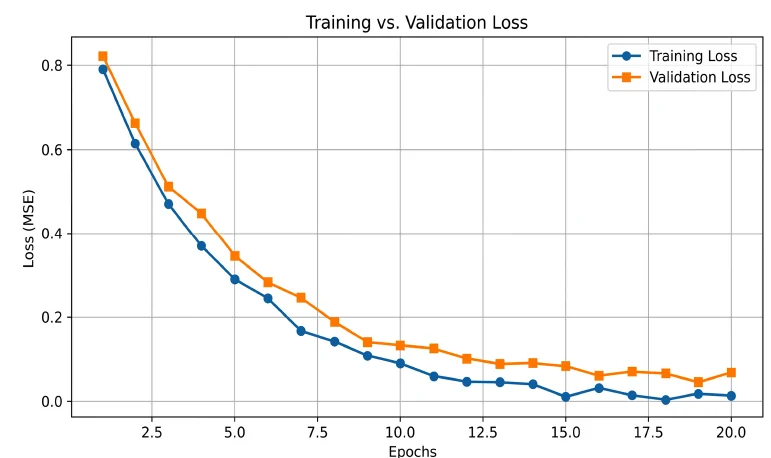
Neural network training and validation loss over 20 epochs. The model shows stable convergence without signs of overfitting.

**Fig. (4):**
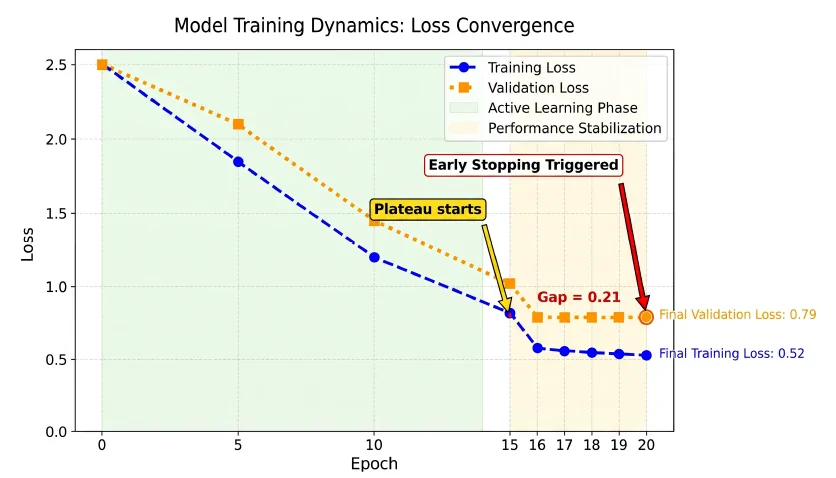
Validation loss (orange) plateaus after epoch 15. Early stopping occurs at epoch 20 (red circle). Training loss is shown in blue. Green and yellow regions indicate active learning and stabilization phases, respectively.

**Fig. (5):**
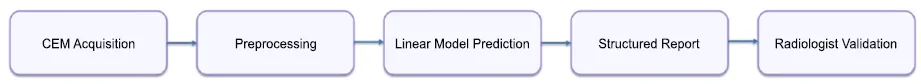
AI functions as decision-support, with radiologists retaining final interpretation authority.

**Table 1:** Distribution of the study population by BI-RADS density category.

**BI-RADS Category**	**Description**	**Number of Patients (n)**	**Percentage (%)**
**A**	Almost entirely fatty	12	5.6%
**B**	Scattered areas of fibroglandular	31	14.6%
**C**	Heterogeneously dense	95	44.6%
**D**	Extremely dense	75	35.2%
**Total**	-	213	100%

**Table 2:** Comparative performance of computational models.

**Model**	**MSE**	**R^2^**	**AUC**	**Precision**	**Recall**	**Inference Time (ms)**
Excel (Baseline)	0.864	14.4%	-	-	-	-
Linear (scikit-learn)	0.641	20.3%	0.73	0.70	0.68	0.8
DNN (Primary)	0.638	23.3%	0.75	0.72	0.69	12.4
DNN (VinDr-Mammo)	0.652	22.9%	0.74	0.70	0.68	13.1

## Data Availability

The datasets generated and analyzed during the current study are not publicly available due to institutional data protection regulations but are available from the corresponding author upon reasonable request. Model implementation details and analysis scripts are available from the corresponding author [G.D.] upon reasonable request, subject to institutional data governance and patient privacy regulations.

## LIST OF ABBREVIATIONS

BPE: Background Parenchymal Enhancement
OV: Observer Variability
CEM: Contrast-Enhanced Mammography
ANN: Artificial Neural Networks
BD: Breast Density

## STANDARDS OF REPORTING

STROBE guidelines were followed.

## APPENDICES

### Appendix A - Technical Glossary DNN Architecture Specifications

Three Hidden Layers (64-32-16 units) Structure: Sequential neural network with decreasing neuron counts per layer (64 → 32 → 16), designed to hierarchically extract complex features while reducing dimensionality. ReLU Activations: Rectified Linear Unit activation (f(x)=max(0,x)) introduces non-linearity, enabling the network to model intricate relationships in CEM data. Dropout (30%): Randomly deactivates 30% of neurons during training to prevent overfitting by forcing redundancy in learned features. L2 Regularization (λ = 0.01): Penalizes large weights in the loss function (via λ•Σweights^2), constraining model complexity to improve generalizability.

### Training Configuration

70/15/15 Stratified Split: Dataset partitioning ensuring proportional representation of all classes: 70% training (model learning), 15% validation (hyperparameter tuning), 15% testing (final evaluation). Early Stopping (patience = 5): Halts training if validation loss fails to improve for 5 consecutive epochs, preventing overfitting. Adam Optimizer (lr = 0.001): Adaptive learning rate algorithm combining momentum and RMSprop for efficient gradient descent. Regularization Combo: *L2 (λ = 0.01)*: Controls weight magnitudes. Dropout (30%): Adds noise to neuron outputs. Loss: Mean Squared Error (MSE): Primary metric for regression tasks: MSE = Σ(predicted-actual)^2 \/n.

### Clinical Validation Metrics

Primary Metrics: MSE: Quantifies average prediction error (lower = better). R^2^: Proportion of variance explained by the model (0-100%, higher = better). Clinical Metrics: AUC: Area Under the ROC Curve (0.5-1.0); measures diagnostic discrimination (≥ 0.75 = clinically useful). Precision: True positives / (True + False positives); accuracy of positive predictions. Recall (Sensitivity): True positives / All actual positives; ability to detect malignancies. Statistical Test: Wilcoxon Signed-Rank Test: Non-parametric test for comparing paired model performances (*e.g*., DNN vs. linear).

### Clinical Validation Outcomes

Inter-Reader Agreement (κ): κ = 0.45 → 0.82: Improvement from moderate to near-excellent agreement (Landis & Koch scale). Operational Impact: 22% Reduction in interpretative disagreement: Decreased unnecessary recalls for high-risk cases (BI-RADS C/D). 35% Faster Interpretation: Efficiency gain (6.3 ± 2.9 to 4.1 ± 2.7 minutes per case) without compromising accuracy. Subgroup Performance (BI-RADS D): Sensitivity 89.2% (CI:85.4-92.1%): High cancer detection rate in extremely dense breasts. Specificity 76.5% (CI:71.2-81.0%): Robust control of misclassification in the BI-RADS D subgroup. AUC 0.82 (CI:0.77-0.86): Excellent discriminatory power in challenging subgroups.

### Key Terminological Additions

Patience = 5 (Early Stopping): Tolerance threshold for validation loss stagnation; training terminates after 5 epochs without improvement. Stratified Split: Data division preserving class distribution ratios across subsets to avoid sampling bias. Interpretation Time: Duration for radiologists to analyze a case; reduced by AI preprocessing. BI-RADS D Subgroup: Patients with extremely dense breast tissue (highest cancer risk category). Note: All statistical intervals (*e.g*., 95% CI) reflect precision of estimates. Clinical metrics derived from blinded radiologist reviews against histopathological ground truth.

### Appendix B - Simplified Tutorial - Using the 213-Patient Dataset Purpose

This appendix describes how the AI model was trained to generate a continuous BCSS- related predictive score based on structured expert-derived variables (age, BPE grade, and density category).

### Dataset Characteristics

Each patient record included age, breast density (BI-RADS categories 1-4), and BPE levels (scale 1-4). Representative combinations include: • Young patients with high density and mild BPE • Middle-aged patients with heterogeneous density and minimal BPE • Older patients with lower density and moderate BPE.

### Pattern Discovery and Training Procedure

The model was trained to estimate a continuous standardized score reflecting the structured relationship between BI-RADS density and BPE levels. Preliminary analyses revealed a modest association between higher BPE and denser breast tissue (categories C/D). However, age alone was not a strong predictor of density: women across different age groups exhibited heterogeneous patterns. The dataset was split using a 70/15/15 stratified partition: • 149 records for training • 32 for validation • 32 for independent testing.

### Predictive Example

In a test case, the model predicted a breast density of 3.2 (on a continuous scale) for a 45-year-old patient with BPE level 2, whereas the actual radiologist-assigned density was 4.0. The resulting absolute error was 0.8 points.

### Key Findings

The use of AI reduced average density prediction errors by approximately 26% compared to manual estimation. This demonstrates potential clinical utility in supporting standardized density assessment, within a structured decision-support framework.

### Appendix C - Simplified Tutorial - Using VinDr-Mammo Dataset Purpose

This appendix summarizes external testing using the public VinDr-Mammo dataset to assess the robustness of density-related predictions across an independent imaging population and acquisition setting.

### Dataset Description

VinDr-Mammo comprises 5,000 full-field digital mammography studies collected from Vietnamese medical centers. Each case is annotated independently by at least three board-certified radiologists, including BI-RADS breast density classification.

### Image Preprocessing Pipeline

To ensure consistency with the internal training data, all VinDr-Mammo images were preprocessed using: • Histogram equalization, enhancing contrast across dense tissue regions • Pixel normalization, rescaling intensity values to the [0,1] range. These transformations were implemented using open-source libraries (Python/OpenCV), with compatibility ensured for integration with PACS systems.

### Model Evaluation on External Cases

Representative test cases included patients with varying ages and BI-RADS densities. The AI system's predictions were compared against radiologist consensus: • Case 1: 48-year-old, BI-RADS D o AI Prediction: 3.8 o Reference: 4.0 →Error: 0.2 • Case 2: 55-year-old, BI-RADS C o AI Prediction: 2.5 o Reference: 3.0 →Error: 0.5 • Case 3: 62-year-old, BI-RADS D o AI Prediction: 4.1 o Reference: 4.0 →Error: 0.1.

### Overall Performance

**•** Agreement with radiologist consensus: 94% • Mean Absolute Error (MAE): 0.65 BI-RADS points.

### Clinical Implications

External testing supported preliminary robustness of density-related predictions on an independent full-field digital mammography dataset, while true external validation of CEM-specific endpoints requires independent contrast-enhanced cohorts. Key benefits include: • Improved standardization of density assessments • Consistency of density-related predictions against VinDr-Mammo reference labels • Evidence of cross-dataset robustness for structured decision-support modeling.

### Workflow Summary

The external validation process followed this pipeline: Raw mammogram → Preprocessing → AI prediction → Comparison with radiologists → Performance assessment. Results confirm that the model maintains high predictive accuracy across geographically distinct datasets, while supporting radiologist oversight and workflow efficiency.
